# Altered Reward Processing System in Internet Gaming Disorder

**DOI:** 10.3389/fpsyt.2020.599141

**Published:** 2020-12-04

**Authors:** Syeda Raiha, Guochun Yang, Lingxiao Wang, Weine Dai, Haiyan Wu, Guangteng Meng, Bowei Zhong, Xun Liu

**Affiliations:** ^1^Key Laboratory of Behavioral Science, Institute of Psychology, Chinese Academy of Sciences, Beijing, China; ^2^Department of Psychology, University of Chinese Academy of Sciences, Beijing, China; ^3^Center for Cognition and Brain Disorders, The Affiliated Hospital of Hangzhou Normal University, Hangzhou, China; ^4^Zhejiang Key Laboratory for Research in Assessment of Cognitive Impairments, Hangzhou, China; ^5^Sino-Danish College, University of Chinese Academy of Sciences, Beijing, China; ^6^Sino-Danish Center for Education and Research, Beijing, China; ^7^CFIN and Pet Center, Aarhus University, Aarhus, Denmark; ^8^Centre for Cognitive and Brain Sciences and Department of Psychology, University of Macau, Taipa, Macau

**Keywords:** internet gaming, addiction, reward processing, stimulus-preceding negativity, feedback-related negativity, impulsivity, ERP, gaming addiction

## Abstract

Converging evidence indicates that addiction involves impairment in reward processing systems. However, the patterns of dysfunction in different stages of reward processing in internet gaming addiction remain unclear. In previous studies, individuals with internet gaming disorder were found to be impulsive and risk taking, but there is no general consensus on the relation between impulsivity and risk-taking tendencies in these individuals. The current study explored behavioral and electrophysiological responses associated with different stages of reward processing among individuals with internet gaming disorders (IGDs) with a delayed discounting task and simple gambling tasks. Compared to the healthy control (HC) group, the IGD group discounted delays more steeply and made more risky choices, irrespective of the outcome. As for the event-related potential (ERP) results, during the reward anticipation stage, IGDs had the same stimulus-preceding negativity (SPN) for both large and small choices, whereas HCs exhibited a higher SPN in large vs. small choices. During the outcome evaluation stage, IGDs exhibited a blunted feedback-related negativity for losses vs. gains. The results indicate impairment across different stages of reward processing among IGDs. Moreover, we found negative correlation between impulsivity indexed by BIS-11 and reward sensitivity indexed by SPN amplitude during anticipation stage only, indicating different neural mechanisms at different stages of reward processing. The current study helps to elucidate the behavioral and neural mechanisms of reward processing in internet gaming addiction.

## Introduction

Internet gaming disorder is a rapidly increasing concern in today's world. It is a preoccupation and obsession with internet games that interferes with one's social, personal, or occupational life, with typical symptoms of dependence being tolerance, withdrawal, and failed attempts to quit the habit (1). As one of the most common behavioral addictions, it is an emerging health concern. It has been included as Internet Gaming Disorder in ICD-11 and as a “Condition for Further Study” in DSM-5. Internet gaming disorders (IGDs) often struggle in their day-to-day activities, relationships, and jobs because of prolonged game play. They are more likely to have poor sleep quality (2), tend to suffer from emotional problems such as depression and anxiety (2, 3), have poor coping skills (4) and are more prone to developing psychopathology or psychopathological symptoms in the long run (5–7). IGDs often use internet games as an escape from negative moods and feelings, such as hopelessness and guilt (1), allowing them to feel relaxed (6) and in control of the situation (8).

Reward processing is an important aspect of human functioning affecting daily life, and has also been regarded as a key neural mechanism involved in behavioral and cognitive processes related to addiction (9, 10). Impairments in reward processing is the core symptom of many kinds of mental and neurological diseases (11, 12), including drug addiction. It can be classified into two stages: reward anticipation and outcome evaluation (13).

Reward anticipation refers to the incentive salience of a reward. Incentive salience is a psychological process that imbues the perception of stimuli with salience and transforms them into incentive stimuli. Previous addiction studies conducted on substance addiction and internet gaming disorder indicated altered reward processing system among the addicts during the reward anticipation stage. They found less activations in ventral stratum and decreased prefrontal cortical sensitivity to monetary rewards (14–17).

Outcome evaluation refers to the hedonic enjoyment received from reward consumption. IGDs have been found to have alterations in the reward processing system (18–20). The addiction studies conducted on substance addiction and behavioral addiction (i.e., internet gaming disorder) have found addicts to be driven toward high rewards and tend to ignore negative consequences, thus resulting in impaired decision making process and risk taking tendencies (21–26).

Monetary rewards are frequently used to study neural mechanisms involved in reward processing among healthy and addicted individuals (27, 28). However, the findings have been inconsistent about whether individuals with addiction have enhanced or blunted responses to monetary rewards.

The inhibitory control dysfunction theory (29) attempts to explain the alterations in the reward processing systems underlying addiction. It proposes impulsivity and reward processing as the underlying factors of addiction that play a role in promoting or limiting drug use at each of the three stages of addiction: (i) initiation of use, (ii) maintenance of use, and (iii) relapse. According to this theory, impulsivity is a personality trait while impairment in reward processing refers to sensitivity to rewards (positive effects of the drug) paired with insensitivity to punishment (negative outcomes of the drug). This theory has often found support from the research studies (30–34) that found addicts to be impulsive and indulge in risky decision making.

Currently, there is no conclusive evidence from previous studies providing a consensus about the neural correlates of the reward processing system at different stages of reward processing. In the current study, we focused on three key ERP components: stimulus-preceding negativity (SPN), feedback-related negativity (FRN), and P300. SPN is a negative-going slow wave. It is considered an electrophysiological index of reward expectation. Previous research has shown that people with substance dependence had larger SPN while anticipating substance related cues than controls (35, 36). The two other ERP components, FRN and P300, play important roles in outcome evaluation. FRN is usually a negative deflection following feedback onset that typically peaks around 250 ms. Previous studies have found that people with substance dependence have a larger FRN peak, indicating impairment in outcome evaluation processes (23, 37). P300 is a positive deflection typically peaking around 300–500 ms after feedback onset. Previous studies have found that people with substance dependence have larger P300 amplitudes than controls, indicating their poor attentional control.

IGDs have been found to have high impulsivity (38) and high sensation seeking (5). These personality traits are associated with inability to delay gratification, leading to steep delay discounting. Delay discounting refers to the subjective devaluation of an outcome with an increase in delay of its attainment (39, 40). Previous studies have found IGDs to be highly impulsive, which is reflected by a dysfunctional prefrontal cortex (41) and decreased frontostriatal connectivity (42), leading to risky decisions.

IGDs have altered risk evaluation, high risk taking tendencies, and tend to indulge in risky decision making (30, 32, 43–45). They were found to have enhanced reward sensitivity and decreased loss sensitivity compared to control counterparts. A similar study conducted on IGD adolescents (46) testing the dual-system model found that individuals with internet gaming addiction have altered reward processing and inhibitory control in a gambling task and a Go/No Go task, respectively. These impairments in reward processing system make it difficult for IGDs to quit playing internet games despite negative effects on their daily life, such as poor grades and deterioration of relationships (5, 7). Their altered reward processing system also makes them prone to developing psychopathology (47, 48).

A few fMRI studies (32, 34, 49) have examined the neural basis of reward processing among IGDs. However, the low temporal resolution made fMRI a less powerful technique to answer the question about different processing stages. Instead, ERP technique has fine-grained temporal resolution, and is uniquely suitable to investigate in detail the time course of reward processing in internet gaming addiction. To our knowledge, no ERP study on internet gaming disorder to date has explored the neural correlates of behavioral addiction across different stages of reward processing. The P300 and FRN components have frequently been studied among IGDs (19, 37). However, the SPN component occurring at the early stages of reward processing often remains a neglected ERP component in these studies. In light of previous work indicating possible abnormal reward system in IGDs, the current study aimed to bridge this gap by exploring alterations in the reward processing system during different stages of reward processing. In the current study, we examined the reward processing systems in IGDs as compared to the HCs while they expected and received rewards during the delayed discounting and a simple gambling task. Behaviorally, we anticipated the IGDs to make more risky choices and discount delays more steeply than the HCs. Neurally, we expected decreased risk sensitivity, indexed by smaller magnitude effect on SPN during the anticipation stage and reduced FRN magnitude during the outcome-appraisal stage of reward processing. Moreover, based on inhibitory control dysfunction theory, we predicted that larger P300 amplitude would be observed on gain trials than loss trials. We hypothesized that IGDs discount delays more steeply on a delayed discounting task and make more risky decisions, irrespective of whether they were in a gain or loss condition. With a delay discounting task, we further explored and established the relationship between delay gratification and risky decision making among IGDs.

## Materials and Methods

### Participants

Thirty-five male adults (age 22.06 ± 3.65) with internet gaming disorder and another 39 age-matched healthy male adults (age 21.95 ± 3.47) in total were recruited in this study. They had either normal or corrected-to-normal vision and self-reported no history of physical disability, chronic physical illness, or neurological or psychiatric problems. The inclusion criteria for IGDs required minimum scores of 50 on the Internet Addiction Test (IAT) (50), and 5 on the DSM Test for Internet Gaming Disorder (51), while for the control group, the scores on both the tests were required to be lower than these thresholds. The IAT (Cronbach's α = 0.93; *r* = 0.46) and DSM Test (Cronbach's α = 0.91; *r* = 0.44) were used to screen the IGDs from the control group. These two tests were intended to measure the effect of Internet use on the individual's daily life and the extent of problems caused by it on daily routine, work, social life, sleep routine, and feelings, in accordance with DSM-5 criteria. In addition, the Alcohol Use Disorder Identification Test [AUDIT, (52)], Beck Depression Inventory [BDI-II, (53)], State-Trait Anxiety Inventory-Trait [STAI-T, (54)], and State-Trait Anxiety Inventory-State (STAI-S) were used to exclude those with alcohol use disorder, depression, and anxiety disorders. Moreover, we used the Barratt Impulsiveness Scale-Version 11 [BIS-11, (55)], Behavioral Inhibition System/Behavioral Activation System [BAS/BIS, (56)], and Sensation Seeking Scale (57) to explore impulsivity, reward systems, and sensation seeking in relation to decision making in IGDs. The two groups were counterbalanced on years of education, with high school as the minimum education level (see Table 1).

**Table 1:** Sample characteristics.

	**HCs**	**IGDs**	***p*-value**
Sample size	39	35	
Age (years)	22.06 ± 3.65	21.95 ± 3.47	0.896
Education	15.04 ± 0.56	14.75 ± 0.59	0.520
IAT	22.56 ± 2.04	65.25 ± 2.17	0.000^***^
AUDIT	0.31 ± 0.73	0.19 ± 0.29	0.504
DSM	0.30 ± 0.22	6.83 ± 0.24	0.000^***^
BDI	4.11 ± 5.75	11.25 ± 8.76	0.013^*^
STAI-S	33.96 ± 9.05	42.83 ± 10.43	0.005^**^
STAI-T	32.37 ± 9.16	37.63 ± 11.58	0.218
**BIS-11**			
Motor	28.71 ± 2.15	35.52 ± 2.36	0.013^*^
Attention	26.98 ± 11.08	29.38 ± 9.84	0.397
Non-Planning	25.95 ± 16.48	35.83 ± 14.02	0.029^*^
**BAS/BIS**			
BAS	42.07 ± 4.92	43.75 ± 5.19	0.167
BASD	12.97 ± 2.46	13.29 ± 2.40	0.809
BASF	15.07 ± 2.13	16.25 ± 2.38	0.019^*^
BASR	14.03 ± 1.73	14.21 ± 1.47	0.569
BIS	15.63 ± 2.38	15.92 ± 2.65	0.528
**SSS**			
Boredom susceptibility	1.83 ± 1.47	2.81 ± 1.88	0.143
Disinhibition seeking	3.59 ± 0.33	3.65 ± 0.34	0.812
Experience seeking	3.92 ± 2.08	4.07 ± 1.60	0.642
Thrill and adventure seeking	4.88 ± 2.52	6.48 ± 2.23	0.039^*^

*IAT, internet addiction test; AUDIT, the alcohol use disorder identification test; DSM, DSM test for internet gaming; BDI, beck depression inventory; STAI-S, state trait anxiety inventory-state; STAI-T, state trait anxiety inventory-trait; BIS-11, barratt impulsiveness scale, version 11; BIS/BAS, behavioral inhibition system/behavioral activation system; BASD, behavioral activation system-drive; BASF, behavioral activation system-fun-seeking; BASR, behavioral activation system-reward; BIS, behavioral inhibition system; SSS-V, sensation seeking scale form V*.

* *p < 0.05*,

** p < 0.01, and ***p < 0.001.

Thirty-three IGDs and 35 HCs completed the delay discounting task, among which 24 IGDs and 26 HCs participated in the ERP study with a simple gambling task. In the gambling task analysis, five subjects (one IGD and four HCs) were excluded from further analysis because they mostly chose one option (high or low, over 90%). With this, we sought to ensure participants' conscious attention on the trials, while employing the excluding criteria comparable to that reported by Dewitt et al. (58). In the ERP analysis, another three subjects (two IGDs and one HC) were excluded from the SPN analysis, and four subjects (two IGDs and two HCs) were excluded from the FRN and P300 analysis, respectively, because too few effective epochs were left after removing artifacts.

The research was approved by the Institutional Review Board of the Institute of Psychology, Chinese Academy of Science before the commencement of the experiments. All participants signed a consent form before participating in the experiment.

### Procedure

#### Delayed Discounting Task

In this task, participants were required to choose between a small gain that was available immediately and a fixed larger gain (¥ 1,000) that was delayed by one of five periods of time (1 week, 1 month, 6 months, 3 years, and 15 years). The participants practiced by making choices between 2-week delayed periods and varied amounts available immediately, to familiarize themselves with the procedure before the formal experiment procedures. During the formal experiment blocks, there were seven choices for the immediate gain amount with five delayed periods: 1 week, 1 month, 6 months, 1 year, and 15 years. An algorithm was used to adjust the amount of the immediate gain across the seven to estimate the subjective values of delayed gains. The participants were given the opportunity to restart the procedure after each trial to avoid errors that could lead to inaccurate estimates of their subjective values, in case they wanted to modify or change their choice.

#### Simple Gambling Task

**Figure 1:**
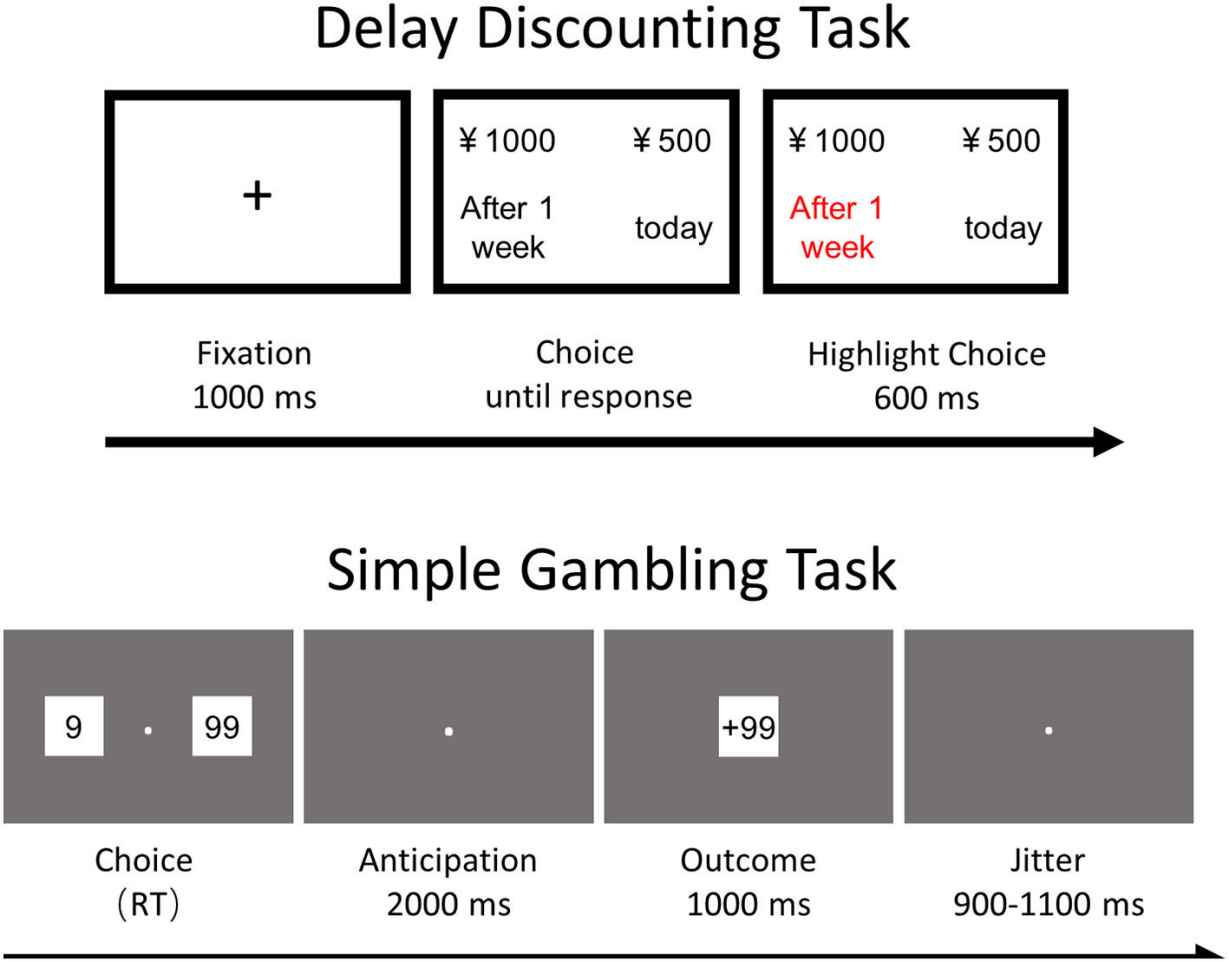
The experiment design of the delay discounting task and the simple gambling task.

In the simple gambling task, the participants were instructed in the beginning that their remuneration for the experiment would be dependent on the amount they won or lost in this task (see Figure 1). The main procedure consisted of one practice block and six main blocks. The practice block consisted of 10 trials, and each main block comprised 80 trials with a short break between two consecutive blocks.

The trial contained two options (9 for low risk and 99 for high risk) appearing on either side of a fixation cross, which disappeared until responses were made. The participants were required to choose one option by pressing “f” (for the left option) or “j” (for the right option). After a response was made, only the fixation cross remained on the screen for 2,000 ms followed by a feedback slide for 1,000 ms. The feedback slide contained points with a “+” or “-” sign to indicate the points they had won or lost for their response.

### Electroencephalographic (EEG) Recording and Preprocessing

We used Brain Products System (64 channel amplifier, Brain Vision Recorder Version 2.0; Brain Products, GmbH, Germany) for EEG data recording. The Easy Cap electrode system (EASY-CAP, Herrsching) was used to place electrodes in accordance with the 10–20 system on 64 positions. Vertical eye movements were recorded by placing one electrode below the right eye (VEOG). The channel FCz was set as the reference channel during data recording. Chloride free-electrolyte gel was used to gently abrade the scalp to keep impedances in electrodes below 5 kΩ. EEG data were recorded at a sampling rate of 500 Hz with a pass band of 0.01–100 Hz.

We adopted the analysis approaches from previous studies (26, 59). EEGLAB toolbox (60) running under MATLAB software was used for the raw data analysis. The data were re-referenced to the average of channels TP9 and TP10. The reference channel FCz was then added back to the data. A low-pass filter of 20 Hz was used to determine the SPN for pre-feedback epochs (2,000 ms pre-stimulus, 500 ms post-stimulus), while a band-pass filter of 0.1–20 Hz was applied for FRN and P300 for post-feedback epochs (200 ms pre-stimulus, 800 ms post-stimulus). The independent component analysis ocular correction method was used to remove any artifacts present due to eye movements and eye blinks in the epochs after visual inspection. We set the activity from −200 to 0 ms, and from −2,000 to −1,800 ms as baseline correction for post-feedback components (FRN and P300), and the pre-feedback component (SPN), respectively.

### Data Extraction

For the delay discounting task, the area under the discounting curves (AUCs) for each subject were calculated with the method in line with previous studies (61–63). The AUC values were used since they are not affected by the quality of fit of the discounting models, and are usually more normally distributed than other discounting function parameters (e.g., k or h values) (64).

For the gambling task, we calculated the effects of valence (gain or loss) on the basic risky choice proportion and conditional risky choice proportions (choice following the previous outcome) and reaction times.

At the EEG level, we recorded the peak amplitudes of the four conditions: high gain, low gain, high loss, and low loss on SPN, FRN, and P300 components. The component values for SPN were measured with four electrodes in the left-hemisphere (C3, C5, FC3, and FC5), and four electrodes in the right-hemisphere (C4, C6, FC4, and FC6) electrodes according to the topographic maps and grand average waveforms. The time window for SPN was observed at −200 to 0 ms (before feedback). The FRN was extracted from 250 to 350 ms after the feedback onset at FCz, Fz, and Cz, where it was observed to be maximal. P300 was measured with CPz and Pz from 350 to 450 ms (after the feedback). The channels and time windows for each component were selected according to the activations on the topographic maps and the peak of the waveform, respectively.

### Statistical Analysis

Two-sample *t*-tests were applied to the AUC values in the delay-discounting task and the basic choice risk proportion. The basic choice reaction time in the gambling task was analyzed with an analysis of variance (ANOVA) of Group (IA group vs. HC group) × Risk (high risk vs. low risk). The conditional analysis was achieved with a three-way ANOVA of Group (IA group vs. HC group) × Risk (high risk vs. low risk) × Previous Outcome (win vs. loss).

The ERP data for the simple gambling task were analyzed twice, one pre-feedback condition for determining the SPN component and one post-feedback stimuli for FRN and P300 components. Repeated-measure ANOVAs were used for the SPN component with the between-subject factor GROUP (HC group vs. IGD group) and within-subject factor Magnitude (high vs. low) and Hemisphere (right vs. left). Repeated-measure ANOVAs were used for FRN and P300 component, with the between-subjects factor Group (HC group vs. IA group) and within-subject factor Magnitude (high vs. low) and another within-subject factor Valence (gain vs. loss). Greenhouse-Geisser correction was used for two or more factors with major effects. *Post-hoc* analysis was conducted using Bonferroni corrections.

## Results

### Demographic and Behavioral Data

Table 1 shows the demographic data for the HC and IGD groups. The groups did not differ in age and educational level. As expected, the groups differed significantly on the IAT and DSM Test for Internet Gaming. Moreover, the IGD group scored higher on BDI, STAI, the Motor subscale, Non-Planning in the BIS-11, and the BAS-Fun-Seeking subscale in the BAS/BIS.

### Delayed Discounting Task

**Figure 2:**
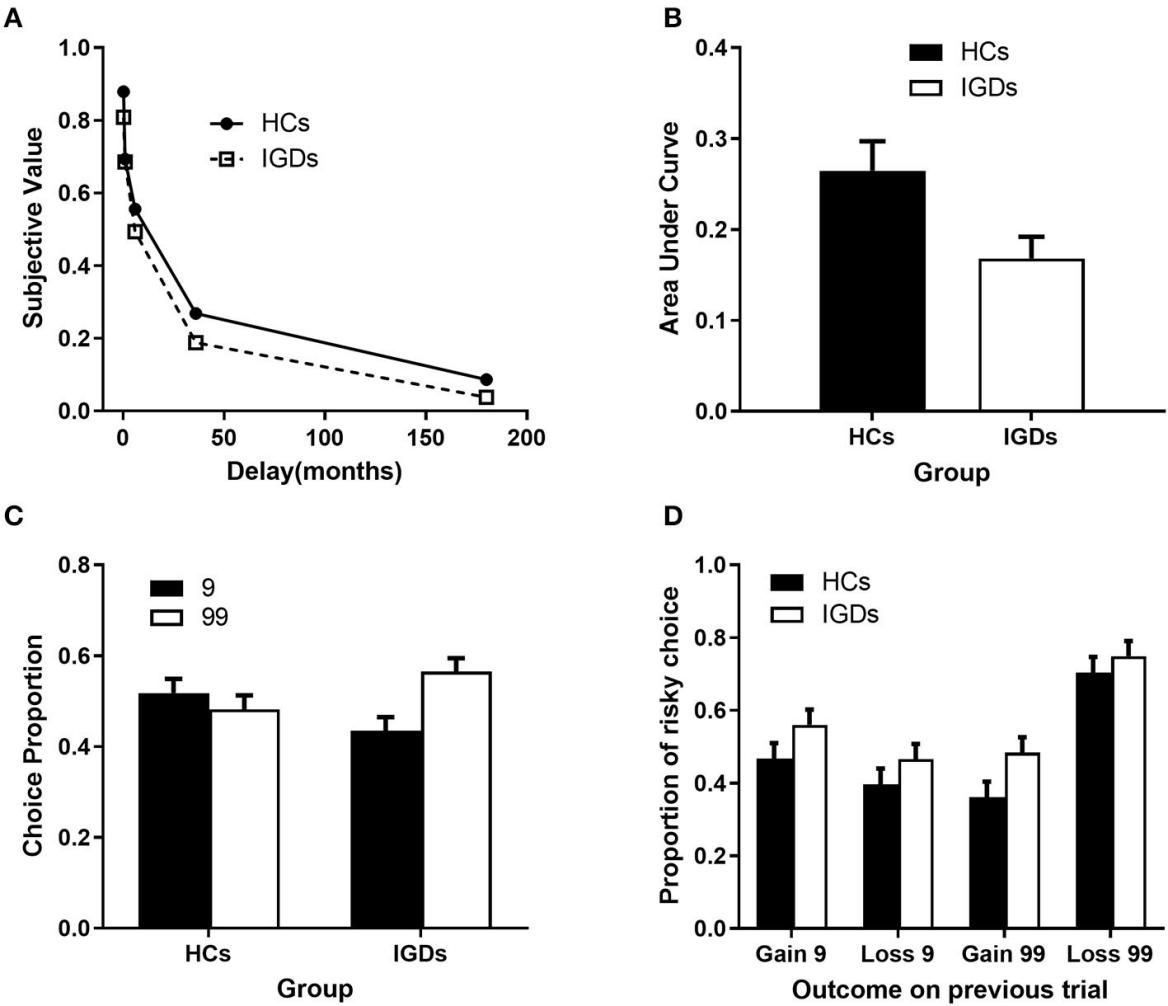
Behavioral Results in delay discounting task and Gambling Task. **(A)** Slope for area under the curve (AUC) and **(B)** the distribution of mean value of area under the curve on Delayed Discounting task. **(C)** Proportion of Basic Choice and Reaction Times on Simple Gambling Task. **(D)** Proportion of Risky Choice on Simple Gambling Task.

For the delay discounting task, there was a significant group effect, *t* (66) = 2.57, *p* = 0.012, indicating that the IGD group discounted delayed outcomes (M_AUC_ = 0.17, SD_AUC_ = 0.02) more steeply than the HC group (M_AUC_ = 0.26, SD_AUC_ = 0.03) (see Figures 2A,B).

### Simple Gambling Task

#### Reaction Time

For decision making time, there was no significant main effect of group, *F*_(1,43)_ = 0.82, *p* = 0.371, η_*p*_^2^ = 0.019, or condition effect, *F*_(1,43)_ = 1.60, *p* = 0.201, η_*p*_^2^ = 0.038, nor a significant condition × group interaction effect, *F*_(1,43)_ = 0.14, *p* = 0.707, η_*p*_^2^ = 0.003.

#### Basic Choice

There was a marginally significant group effect, *t* (43) = 1.82, *p* = 0.076, indicating that a higher proportion (56.5%) of the IGD group preferred risky choices than the HC group (48.2%). Specifically, IGDs tended to make more risky decisions than the chance level (50%), *t* (22) = 2.020, *p* = 0.056. In contrast, HCs exhibited a risk-neutral pattern, *t* (21) = −0.563, *p* = 0.579 (see Figure 2C).

#### Risky Choice

Both groups tended to risk larger amounts after facing a loss in the previous trial than when they had a gain in the previous trial, *F*_(1,43)_ = 9.59, *p* = 0.003, η_*p*_^2^ = 0.182, and after making a high-risk choice in the previous trial than when they made a low-risk choice in the previous trial, *F*_(1,43)_ = 21.38, *p* = 0.000, η_*p*_^2^ = 0.332. The IGD group made more risky choices than the HC group, irrespective of the previous outcome, *F*_(1,43)_ = 6.12, *p* = 0.017, η_*p*_^2^ = 0.125 (see Figure 2D).

### ERP Results

#### FRN

**Figure 3:**
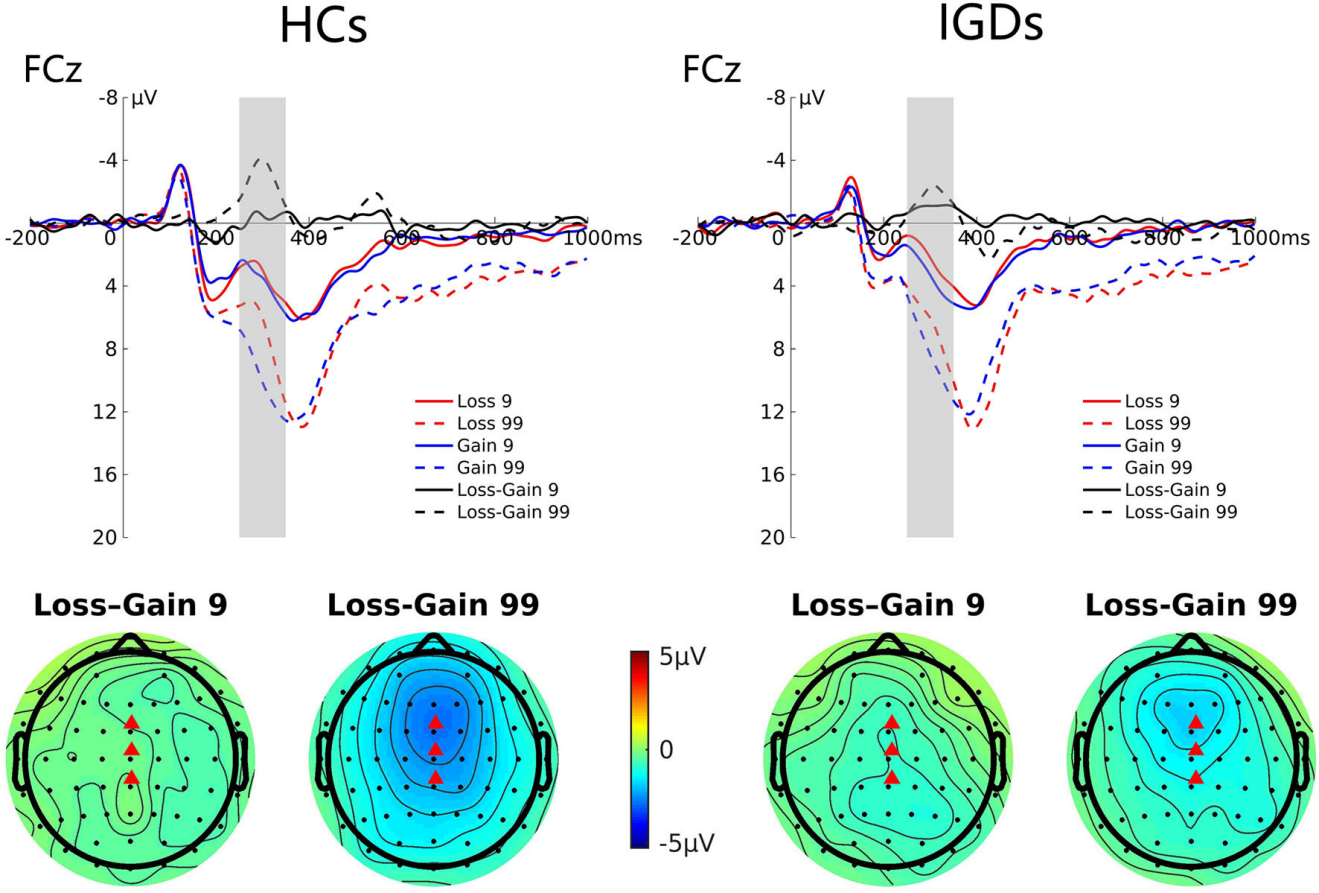
Grand average ERP waveforms following low- and high-risk decisions for HCs and IGDs at FCz. The upper figure shows the ERP waveforms for HCs and IGDs at FCz. FRN is calculated as the difference between loss and gain waveforms after feedback, and the time window was depicted as the shaded areas. The lower figure shows the topographic maps on time window 250–350 ms.

Figure 3 presents the grand average ERP waveforms at FCz elicited by gains and losses and their differences, and the topographic map for these two groups. Repeated-measures ANOVA revealed a significant magnitude effect on the FRN component, *F*_(1,41)_ = 12.33, *p* = 0.001, η_*p*_^2^ = 0.231, indicating that the FRN amplitude was higher in high-risk than in low-risk outcomes (−2.11 μV vs. −0.67 μV). The interaction between magnitude and group was also statistically significant, *F*_(1,41)_ = 5.17, *p* = 0.028, η_*p*_^2^ = 0.112. Simple effect analysis revealed that the FRN amplitude was greater in high-risk outcomes compared to low-risk outcomes only in the HC group (−2.74 μV vs. −0.36 μV, *p* = 0.000), but not in the IGD group (−1.48 μV vs. −0.97 μV, *p* = 0.392).

#### P300

**Figure 4:**
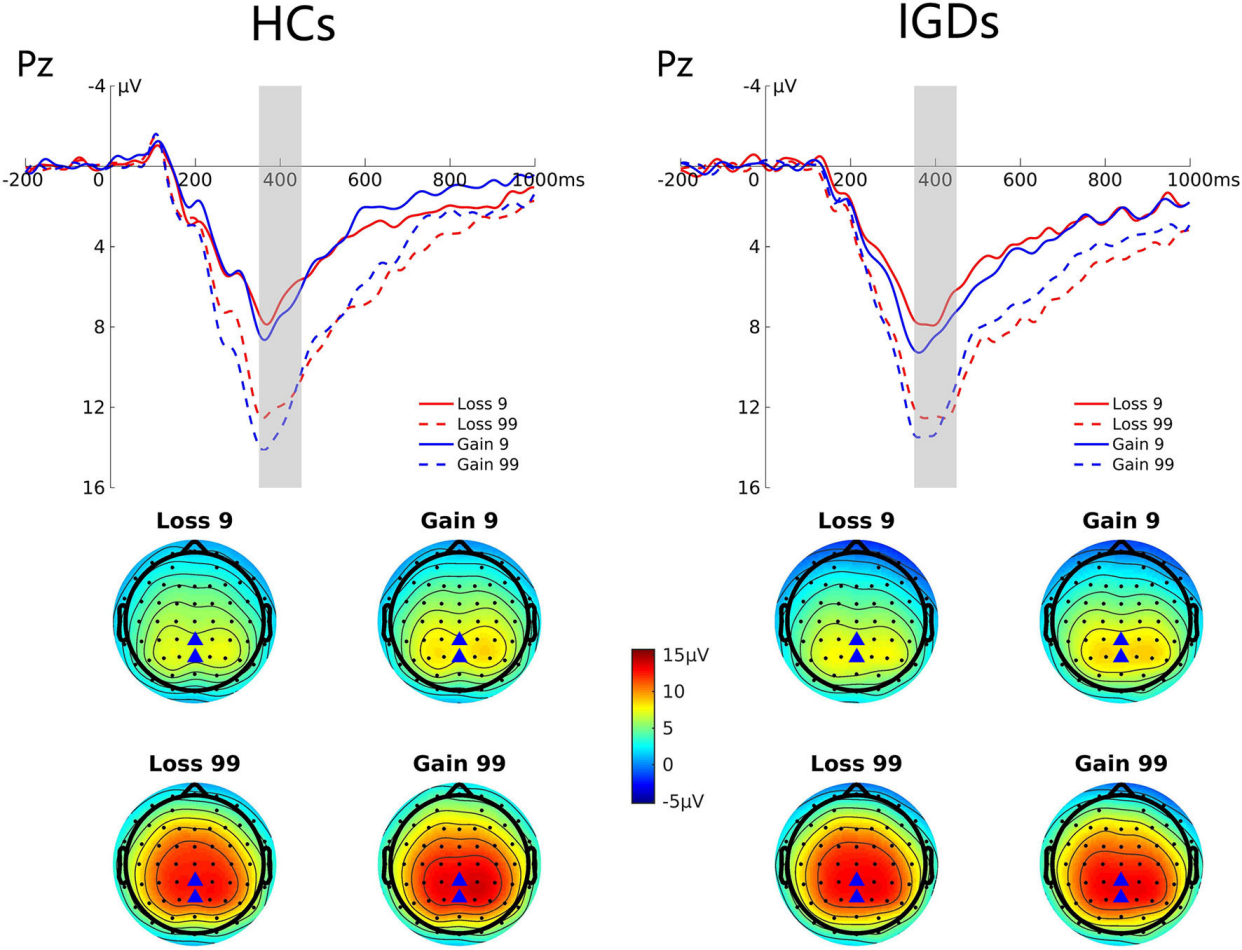
Grand average ERP waveforms following low- and high-risk decisions for HCs and IGDs at Pz. The upper figure shows the ERP waveforms for controls and IGDs at Pz. The time window was depicted as the shaded areas. The lower figure shows the topographic maps on time window 350–450 ms.

Figure 4 presents the grand average ERP waveforms at Pz elicited by gains and losses, and the topographic map for these two groups. Repeated-measures ANOVA revealed significant magnitude effect, *F*_(1,41)_ = 74.47, *p* = 0.000, η_*p*_^2^ = 0.645, indicating that the P300 amplitude was higher in high-risk outcomes than in low-risk outcomes (12.74 μV vs. 7.39 μV); and significant valence effect, *F*_(1,41)_ = 7.51, *p* = 0.009, η_*p*_^2^ = 0.155, indicating that the P300 amplitude was higher in a gain context than in a loss context (10.44 μV vs. 9.70 μV).

#### SPN

**Figure 5:**
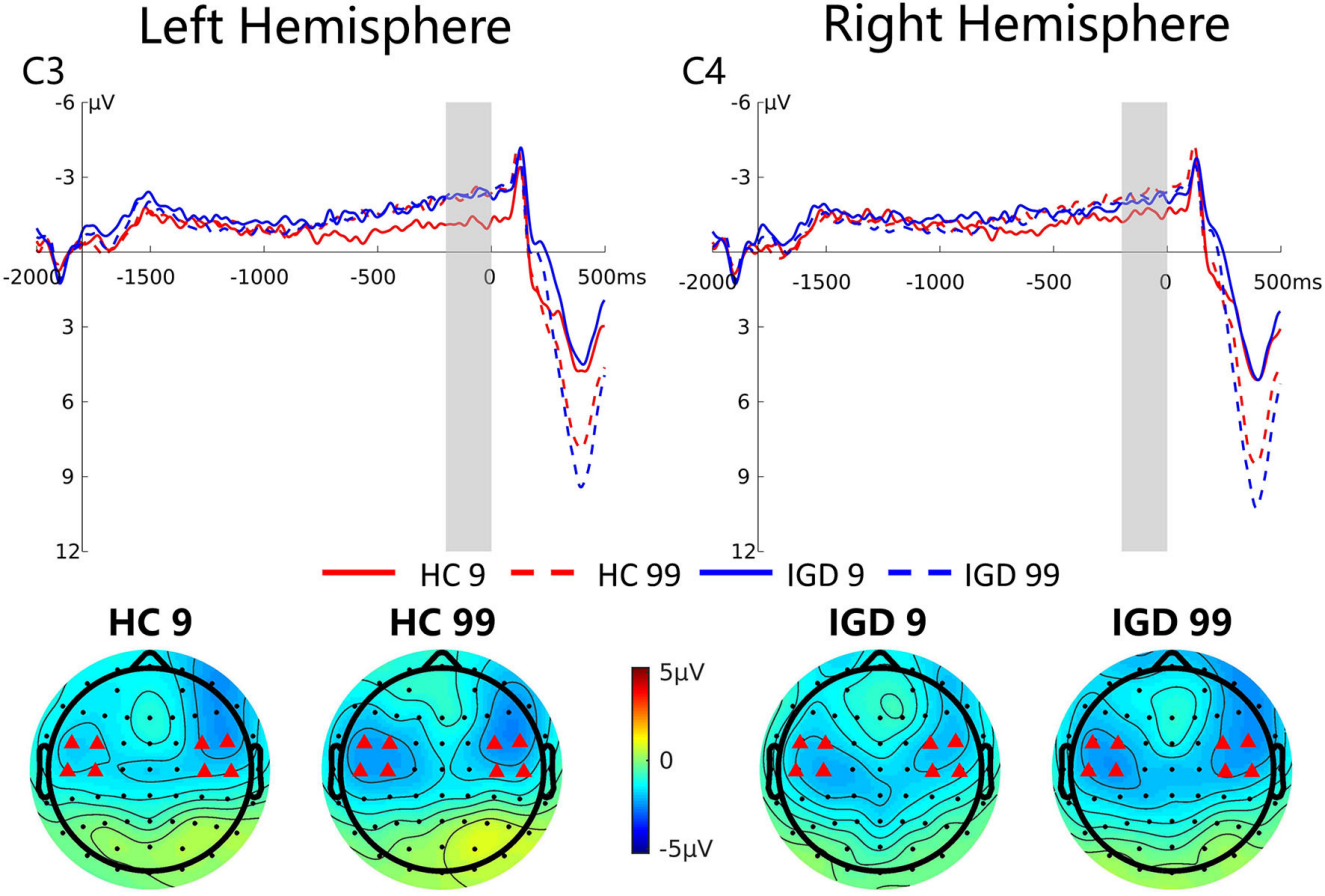
Grand average ERP waveforms following low- and high-risk decisions for HCs and IGDs at C3 and C4. The upper figure shows the ERP waveforms for controls and internet gamer at C3 and C4. The time window was depicted as the shaded areas. The lower figure shows the topographic maps on time window −200 to 0 ms.

Figure 5 presents the grand average ERP waveforms at C3 and C4 and topographic maps of the SPN (−200 to 0 ms) for these two groups. Repeated-measures ANOVA revealed a significant magnitude effect on the SPN component, *F*_(1,42)_ = 5.06, *p* = 0.030, η_*p*_^2^ = 0.108, indicating that the SPN amplitude was higher for high-risk choices than for low-risk choices (−2.26 μV vs. −1.69 μV). The interaction between magnitude and group was marginally significant, *F*_(1,42)_ = 3.03, *p* = 0.089, η_*p*_^2^ = 0.067. Simple effect revealed that the SPN amplitude was greater in high-risk decision making compared to low-risk decision making only in the HC group (−2.41 μV vs. −1.39 μV, *p* = 0.006), but not in the IGD group (−2.12 μV vs. −1.97 μV, *p* = 0.727).

### Correlation Results

**Figure 6:**
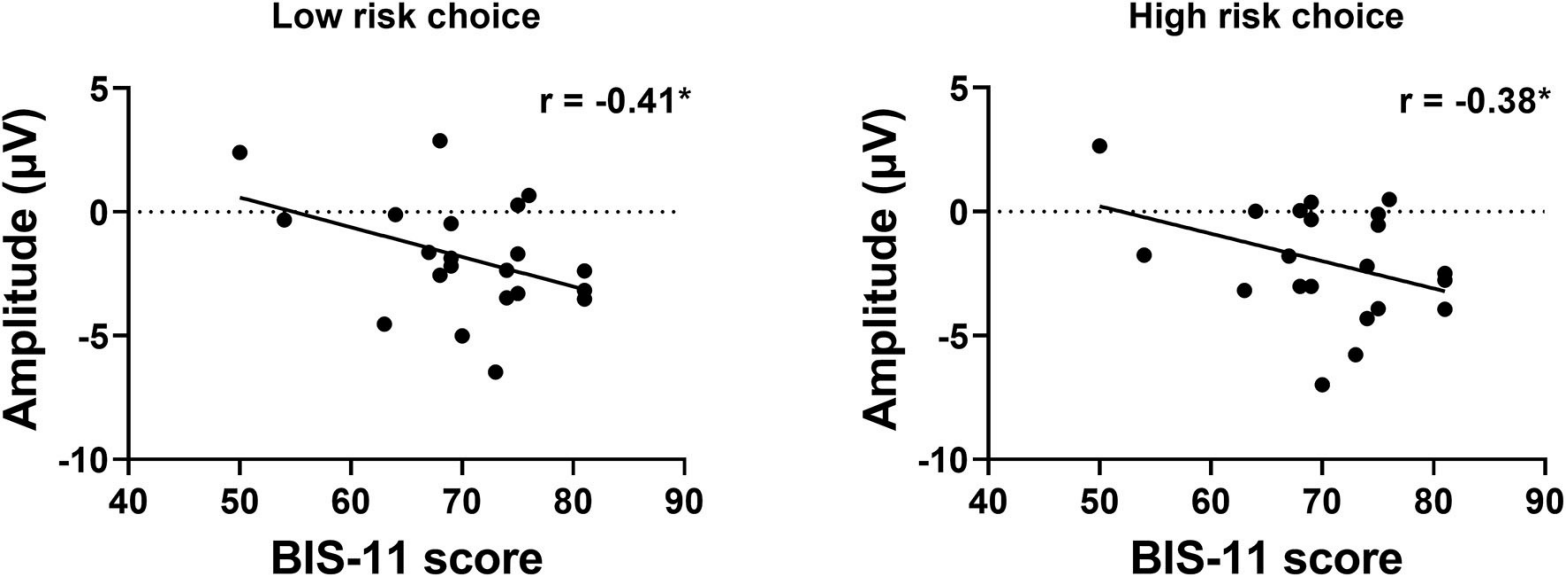
Correlation between BIS score and the SPN amplitude. The left panel shows the result when choosing low risk choices, and the right panel shows the result when choosing high risk choices.

To examine the potential relationship between impulsivity and risk-taking tendencies at the individual difference level, we calculated the correlation between the impulsivity indices (i.e., AUC and BIS score) and basic choice, risky choice, and three ERP amplitudes within each group independently. Although none of these indices significantly correlated with AUC results, we found significant negative correlation between the BIS score and the SPN amplitude in the left hemisphere when choosing low risk choice, *r* = −0.41, *p* = 0.031, as well as when choosing high risk choice, *r* = −0.38, *p* = 0.044 (see Figure 6).

## Discussion

In our sample of participants, we found that IGDs had relatively higher impulsivity, higher proneness to risky decision making, reduced ability to delay gratification, reduced ability to evaluate risk, and different outcome expectancies in risky situations. The behavior of IGDs, that is, making more risky choices, is supported by neural patterns indicating higher sensitivity to rewards and lower sensitivity to punishment among IGDs.

Previous research on substance addiction found that people with substance dependence discounted delayed gains more steeply than non-dependent people on a delay discounting task (65–68). This effect has been found to hold true for IGDs, which were shown to be unable to delay gratification, as indicated by their steep pattern of discounting delayed gains on a delayed discounting task (41, 61, 69). The results of the delay discounting task are supported by the high impulsivity scores on the BIS among the IGD group compared to the HC group. The IGD group were more impulsive on the motor impulsiveness and non-planning subscales than the HC group, in accordance with previous studies (70–73). However, there were no differences in attention impulsivity subscale of BIS-11 between the two groups. This may be attributed to the positive effects of online gaming or video gaming on individuals in increasing sustained attention (74, 75). IGDs were revealed to be more thrill and adventure seeking and more sensitive to rewards than HCs. These observations are in line with the findings of previous studies on problematic internet gaming (76–78).

On the behavioral level, risk-taking tendencies were more pronounced in the IGD group. They were also found to be more prone to make risky choices, irrespective of whether the previous outcome was a win or a loss. These results are in accordance with previous literature that found problematic IGDs to be more focused on and sensitive to wins and less focused on and less sensitive to losses (79, 80). The enhanced reward sensitivity and decreased sensitivity to losses lead them to risky decision making. Previous studies have indicated an association between risky choices and personality factors as impulsivity, sensation seeking, thrill seeking behaviors among addicts. They found that addicts tend to ignore the negative consequences of the situation and focus only on positive rewards (5, 43, 44, 46). Consistently, the IGD group in our current study were also found to have enhanced sensitivity toward rewards as indicated by their higher scores on the BAS/BIS and thrill seeking than the HC group, as well as their less sensitivity to loss, indexed by behavioral choices, SPN and FRN amplitudes in simple gambling task.

At the early stage of reward processing, the SPN was more negative for the larger risk than the smaller risk for the HC group, while no significant differences were found in the IGD group. These results indicated that IGDs expected the same reward outcome whether the risk was high or low, but HCs expected more on a larger risk. Furthermore, the IGDs were less concerned about the outcome, indicating their high risk-taking tendencies. These results are consistent with previous findings that found an altered ability to evaluate risk among IGDs (22, 32, 81).

At the later stages of reward processing, the more prominent FRN amplitude for high-risk choices in comparison with low risk choices was observed in the HC group. However, such results diminished in the IGD group, indicating their increased risk-taking tendencies and decreased sensitivity toward high risk situations. FRN is associated with the binary evaluation of positive vs. negative outcomes (gains vs. losses in our study) based on external feedback that outcome is worse than expected (82). The amplitude of FRN increases when external feedback indicates a negative outcome (i.e., loss). However, the previous studies found that IGDs are less sensitive to negative outcomes. Our results of FRN are consistent with previous studies on individuals with internet gaming disorder (23, 37) that found IGDs to have reduced amplitudes on FRN than healthy controls. Similar to our results of the FRN component, these studies also found a blunted risk effect among IGDs irrespective of the feedback response. Our results on FRN seems to be comparable to the previous studies have found blunted FRN to be associated with unplanned impulsivity and high scores on BIS/BAS system (83, 84). In our sample of participants, the amplitude of the P300 component was significantly larger for the gain than the loss condition indicating their attentional allocation to gains more than the losses. This is consistent with previous studies on decision making and risk-taking (85–87) that found an increase in the amplitudes of the P300 component on gain than loss conditions in a gambling task. The P300 component is often regarded as an index of attentional allocation to task relevant stimuli (88). In contrast to previous studies (37, 89), we did not find a significant group effect on P300 amplitude. A possible explanation for this inconsistency that monetary rewards may not be a strong reinforcer for our IGD group. The enhanced preference toward rewards and less sensitivity toward punishment is indicated but monetary rewards may not be a stronger reinforcer for the IGDs than the HCs, resulting in this inconsistency. Previous studies have also indicated mixed findings while using monetary rewards. In severely addicted individuals, such as cocaine addicts, monetary rewards may not elicit the same sensitivity as using the substance reward they are addicted to (14). This may also be explained considering previous studies (90) that reported an improvement in attentional control as a result of playing computer games.

The results of the current study on IGD are very similar to the previous study conducted on IGD of adolescents (46) that also found IGD to depict high impulsivity on BIS-11, greater tendency to novelty seeking experience on BAS-F subscale of BAS/BIS and significantly greater tendency to indulge in thrill and sensation seeking activities than the non-IGDs. They also found IGD to be more prone to making risky choices but could not find any interaction effect between high risky choices and reaction times, in line with the current study. Although we could not find enhanced FRN for the controls directly, we did find the peak amplitude of the difference between loss and gain to be significantly enhanced on FRN, similar to this study. However, they also only found significant magnitude and valence effect on P300 with no interaction effect between group and the peak amplitude on P300, similar to our study. These similarities give an indication that impairment in reward processing is extended to the adulthood following the same pattern as observed among the adolescents suffering from internet gaming disorder.

We found SPN component and subjective impulsivity, as indicated by BIS-11 scores, to be negatively correlated with each other in the IGD group. It indicates that risk sensitivity during the anticipation stage decreases as the impulsivity level increases among IGDs. This is in line with the addiction studies that found high risk taking tendencies to be associated with high impulsivity (91, 92). However, the similar pattern could not be found in the outcome-appraisal stage, nor any correlation pattern could be determined between impulsivity and delayed discounting and risk-taking strategies on the behavioral level. In agreement with studies (93, 94) that indicated that impulsivity and risky decision making are distinct constructs, our results are consistent with the notion that the relation between impulsivity and risk taking is more complex and these personality measures may function as distinct constructs among IGDs (93). The dominant construct in each internet gamer may vary from individual to individual, for example, some IGDs may be impulsive but not risk-taking and vice versa. In a recent study, researchers found impulsivity and risk-taking tendencies to be distinct constructs associated with separate moods. Risky decision making and high-risk behaviors were found to be influenced by the positive emotions while high impulsivity was found to be associated with negative emotions (94). These results indicate the contrast between anticipation and outcome-appraisal stage, in relation to association between decreased risk sensitivity and impulsivity traits. Future studies may explore the effect of each dominant construct on reward processing among IGDs with different degrees of dependence with larger sample sizes.

This study explored the neural correlates of internet gaming disorder across different stages of reward processing. The results strengthen the notion that IGDs share common patterns of reward processing impairments with people with substance dependence. It also gives an insight into the distinct attentional allocation patterns found among IGDs, owing to their gaming addiction. The common and distinct patterns provide useful behavioral and neurological markers for subsequent prevention and intervention.

Due to the high prevalence of internet gaming addiction among young male adults, we selected only young male adults under 30 years old, which may also be one of the limitations of our study. Another limitation is the relatively small sample size. Although our study has indicated the patterns of impairment across different stages of reward processing, larger sample size studies may be required to confirm this impairment pattern, as well as across different age groups. An additional concern is the possible confounding effect of the relatively higher BDI and STAI-S scores of IGDs than HCs, which may bias the results of this study. Here, we would like to mention that the cognitive effects of internet gaming may be manifold and might also be linked to certain factors affecting the decision of the participants, such as mood, emotion and attention. Nevertheless, the results of the two tasks (delayed discounting task and simple gambling task) provide insight into some behavioral and neural patterns of reward processing among IGDs. Similar to people with substance dependence, the internet addiction group showed high impulsivity, reduced ability to delay gratification, altered ability to evaluate risk, altered outcome expectancies from risky situations and decisions, and risk-taking tendencies. However, IGDs in our sample were found to use an avoidance system in response to punishment, giving us an insight into a distinct pattern of reward processing among IGDs. They were found to be much more focused on larger gains and demonstrated less sensitivity to losses.

## Data Availability Statement

The raw data supporting the conclusions of this article will be made available by the authors, without undue reservation.

## Ethics Statement

The studies involving human participants were reviewed and approved by the Review Board of Institute of Psychology, CAS. The patients/participants provided their written informed consent to participate in this study.

## Author Contributions

SR: data curation, writing—original draft, and writing—editing. GY: data curation, writing—original draft, and writing—editing. LW: writing—review and editing. WD: writing—review and editing. HW: writing—review and editing. GM: writing—review and editing. BZ: writing—review and editing. XL: conceptualization, writing—review and editing, supervision, project administration, and funding acquisition. All authors contributed to the article and approved the submitted version.

## Conflict of Interest

The authors declare that the research was conducted in the absence of any commercial or financial relationships that could be construed as a potential conflict of interest.
